# Food Industry By-Products as a Sources of Phytochemical Compounds

**DOI:** 10.3390/foods11121724

**Published:** 2022-06-13

**Authors:** Drago Šubarić, Stela Jokić

**Affiliations:** Faculty of Food Technology, Josip Juraj Strossmayer University of Osijek, 31000 Osijek, Croatia; drago.subaric@ptfos.hr

Phytochemicals, or phytonutrients, are a group of biologically active substances from plants. They have a functional value for the human body, protecting against and preventing disease. So far, over a thousand different phytochemicals have been identified in different plants and their fruits. Modern research aims to identify and explain the possible benefits of these compounds for human health, and also aims to isolate them. For extraction and isolation, innovative green extraction methods are currently an attractive research topic in the areas of food technology, biotechnology, nutrition, the pharmaceutical and cosmetic industries, applied chemistry, etc. Therefore, it is very important to find the most efficient method for the extraction of certain phytochemicals from selected sources [1,2].

Large quantities of solids are created during the processing of raw materials from plants in the food processing industry every year, and their storage, processing, and management present serious ecological and economic problems. The large quantities of by-products created every day are mostly managed through disposal sites, or by making cattle fodder. Therefore, every industry has the goal of optimizing their raw material use in the production process while creating as little waste as possible. Ultimately, waste should not be perceived and treated as “waste”, but as a by-product or raw material for a future process. There is great interest in using by-products from the food industry for various purposes because they contain many potentially useful substances/phytochemicals, and they could represent significant raw materials in the production/development of new products [3].

In the present collection covers mainly scientific topics related to the valorization of food waste and/or food industry by-products, and innovative methods for the extraction of phytochemicals from food/agricultural waste and by-products. An efficient utilization and valorization of mandarin peel is investigated using innovative and green extraction techniques in study by Šafranko et al. [4]. The first step included the extraction and analysis of volatile compounds by performing a supercritical CO_2_ extraction and, after SC-CO_2_ treatment, the exhausted citrus peel waste enriched with bioactive compounds was subjected to subcritical water extraction (SWE) in order to obtain bioflavonoids. Another publication [5] is related to other innovative extraction techniques such as high-voltage electrical discharges (HVED), pulsed electric field (PEF), and ultrasound-assisted extraction (UAE) in order to obtain individual polyphenolic compounds of blueberry pomace extract. These two papers demonstrate that fruit and vegetable by-products are very interesting sources of natural polyphenolic compounds, and extraction from agro-food by-products is considered to be the best approach for their valorization.

In the paper published by Šavikin et al. [6], the established procedure for pomegranate peel valorization and the attainment of stable extract with preserved bioactive compounds is given. The applied technology involved spray-drying with carbohydrate-based (maltodextrin) and protein-based (whey protein) carrier materials in different concentrations. Overall, the results demonstrated that carbohydrate-based microencapsulation can be utilized efficiently for the protection of powder stability and phytochemical characteristics.

Jozinović et al. [7] demonstrate that food industry by-products can be used as raw materials in the production of value-added corn snack products. The addition of investigated by-products (brewer’s spent grain, sugar beet pulp, and apple pomace) significantly improved the nutritional value of the corn snack products (produced in a laboratory single-screw extruder) in terms of increasing the amount of dietary fiber, total polyphenol content, and antioxidant activity.

For the first time, Cvetković et al. [8] studied the nutritional, chemical and sensory quality of oil obtained from the seeds of Podravka and Slavonka varieties of pepper (*Capsicum annuum* L.) by two green extraction methods (cold pressing and supercritical CO_2_ extraction) at laboratory level. Podravka and Slavonka are among the first Croatian varieties of pepper with UPOV (International Union for the Protection of New Varieties of Plants) certificates. The results of this work could contribute to the growing demand for recycling food by-products into new added-value products, helping food producers to achieve higher efficiency in production through the development of innovative methods, new ingredients, food products, and culinary applications.
